# Phosphate position is key in mediating transmembrane ion channel TMEM16A–phosphatidylinositol 4,5-bisphosphate interaction

**DOI:** 10.1016/j.jbc.2022.102264

**Published:** 2022-07-14

**Authors:** Maiwase Tembo, Rachel E. Bainbridge, Crystal Lara-Santos, Kayla M. Komondor, Grant J. Daskivich, Jacob D. Durrant, Joel C. Rosenbaum, Anne E. Carlson

**Affiliations:** Department of Biological Sciences, University of Pittsburgh, Pittsburgh, Pennsylvania, USA

**Keywords:** chloride channel, phospholipid, calcium, patch clamp, *xenopus*, oocyte, transmembrane member 16A, anion channel, signal transduction, cRNA, circular RNA, diC8-PI3P, dioctanoyl 3-monophosphate, diC8-PI4P, dioctanoyl 4-monophosphate, diC8-PI5P, dioctanoyl 5-monophosphate, diC8-PI(3,4)P_2_, dioctanoyl phosphatidylinositol 3,4-bisphosphate, diC8-PI(3,5)P_2_, dioctanoyl phosphatidylinositol 3,5-bisphosphate, diC8-PI(4,5)P_2_, dioctanoyl phosphatidylinositol 4,5-bisphosphate, diC8-PIP_3_, dioctanoyl phosphatidylinositol 3,4,5-trisphosphate, HSD, honestly significant difference, OR2, oocyte Ringer's solution 2, PIP3, phosphatidyl 3,4,5-trisphosphate, TEVC, two-electrode voltage-clamp, TMEM16A, TransMEMbrane 16A, VSP, voltage-sensing phosphatase, Xl-VSP, *Xenopus laevis* voltage-sensing phosphatase

## Abstract

TransMEMbrane 16A (TMEM16A) is a Ca^2+^-activated Cl^−^ channel that plays critical roles in regulating diverse physiologic processes, including vascular tone, sensory signal transduction, and mucosal secretion. In addition to Ca^2+^, TMEM16A activation requires the membrane lipid phosphatidylinositol 4,5-bisphosphate (PI(4,5)P_2_). However, the structural determinants mediating this interaction are not clear. Here, we interrogated the parts of the PI(4,5)P_2_ head group that mediate its interaction with TMEM16A by using patch- and two-electrode voltage-clamp recordings on oocytes from the African clawed frog *Xenopus laevis*, which endogenously express TMEM16A channels. During continuous application of Ca^2+^ to excised inside–out patches, we found that TMEM16A-conducted currents decayed shortly after patch excision. Following this rundown, we show that the application of a synthetic PI(4,5)P_2_ analog produced current recovery. Furthermore, inducible dephosphorylation of PI(4,5)P_2_ reduces TMEM16A-conducted currents. Application of PIP_2_ analogs with different phosphate orientations yielded distinct amounts of current recovery, and only lipids that include a phosphate at the 4′ position effectively recovered TMEM16A currents. Taken together, these findings improve our understanding of how PI(4,5)P_2_ binds to and potentiates TMEM16A channels.

The broadly expressed Ca^2+^-activated Cl^−^ channel TransMEMbrane 16A (TMEM16A) regulates diverse physiologic processes, including contraction of arterial smooth muscle (1, 2, 3), transduction of sensory signals (4, 5), mucosal secretion (6, 7), peristalsis of the gastrointestinal tract (8, 9), and fertilization (10, 11). TMEM16A dysregulation is associated with diseases, such as hypertension (1, 12) and cancers of various tissues (*e.g.*, the breast (13) and pancreas (14)). Owing to its original identification as a cancer biomarker, TMEM16A is also known as Discovered On Gastrointestinal Stromal Tumors protein 1 (DOG1), Oral Cancer Overexpressed 2 (ORAOV2), and Tumor Amplified and Overexpressed 2 (TAOS-2).

Since the original characterization of TMEM16A as a Cl^−^ conducting channel in 2008 (15, 16, 17), substantial progress has been made in determining its structural and biophysical properties. TMEM16A channels are homodimers with each independently operating subunit comprising 10 transmembrane domains with large intracellular N and C termini (18, 19). Transmembrane domains 3 to 7 comprise the Cl^−^ conducting pore (20, 21), and five conserved acidic amino acids from transmembrane domains 6 to 8 coordinate two Ca^2+^ ions (18, 22, 23). The channel gates differently depend on whether one or two Ca^2+^ ions occupy the binding site (24). The Ca^2+^-binding site is embedded in the plasma membrane electric field, giving rise to the weak voltage dependence of TMEM16A gating at intracellular Ca^2+^ concentrations below saturation (18, 22, 23).

In addition to intracellular Ca^2+^, TMEM16A also requires the acidic phospholipid phosphatidylinositol 4,5-bisphosphate (PI(4,5)P_2_) in order to transition to the open conformation (25, 26, 27, 28, 29). Like the currents of other channels potentiated by PI(4,5)P_2_ (30), TMEM16A currents recorded using the inside–out configuration of the patch-clamp technique rundown over time, even with continued application of intracellular Ca^2+^. For many channels, a few basic amino acids at the plasma membrane–cytoplasm interface enable electrostatic interactions with PI(4,5)P_2_, which differs from other highly structured PI(4,5)P_2_-binding sites (*e.g.*, the pleckstrin homology domain, a common structural motif containing more than 100 amino acids (30)). Results from several groups suggest that TMEM16A–PI(4,5)P_2_ binding is electrostatic and relies on the negative charge carried by the PI(4,5)P_2_ phosphates. Indeed, neutralization of six positively charged arginine and lysine residues clustered near the cytoplasmic interface of transmembrane domains 3 to 5 was shown to speed TMEM16A desensitization (26). Another group identified three discrete TMEM16A PI(4,5)P_2_-binding sites that mediate interactions with phosphate groups as well as the membrane-embedded fatty acid tail (29).

Here, we sought to uncover the molecular determinants of PI(4,5)P_2_ interactions with TMEM16A channels. We made electrophysiology recordings of African clawed frog (*Xenopus laevis*) oocytes, which endogenously and abundantly express TMEM16A channels (16). Using a combination of excised inside–out patch clamp along with two-electrode voltage-clamp (TEVC) recordings, we found that TMEM16A channels are regulated by PI(4,5)P_2_ in both the excised patch and whole cell. In excised patches, TMEM16A-conducted currents rundown following patch excision and can be recovered with the application of water-soluble PI(4,5)P_2_ analogs. The extent of recovery depended on the phosphate positions on the inositol ring, revealing a prominent role for the phosphate at the 4′ position. Moreover, we found that removing the 5′ phosphate with a voltage-sensing phosphatase (VSP) did not significantly speed rundown. Together, these findings establish that the inositol-ring phosphate at the 4′ position is key for PI(4,5)P_2_ regulation of TMEM16A channel activity.

## Results

### TMEM16A currents recorded from excised inside–out patches rundown and are recovered by PI(4,5)P_2_

**Figure 1:**
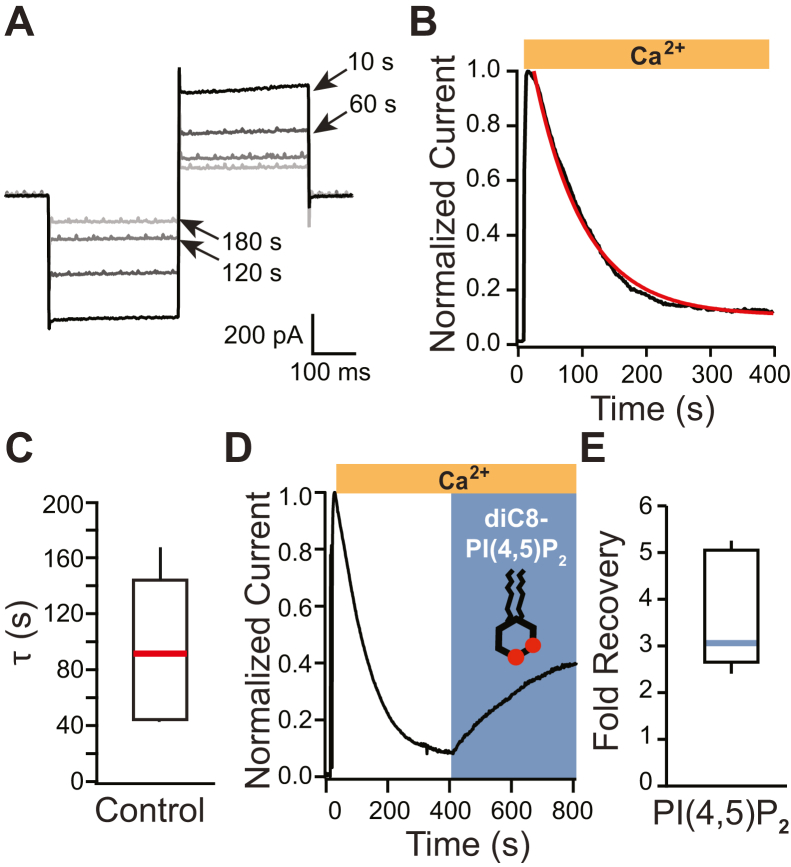
**TMEM16A Ca**^**2+**^**-evoked Cl**^−^**currents rundown in excised patches and are recovered by a diC8-PI(4,5)P**_**2**_**application.***A*, example currents recorded at the indicated times during 150 ms steps to −60 and +60 mV, recorded using excised inside–out macropatches from *Xenopus laevis* oocytes. *B*, normalized plot of current measured at −60 mV *versus* time, fit with a single exponential (*red line*). *C*, box plot distribution of the rate of current decay (τ), measured by fitting plots of relative current *versus* time with single exponentials (N = 11). The *central line* denotes the median, the *box* denotes 25 to 75% of the data, and the *whiskers* represent 10 to 90% of the data. *D*, a soluble synthetic analog of PI(4,5)P_2_, diC8-PI(4,5)P_2_, was applied to excised inside–out patches once current had stably rundown. Currents were recorded at −60 mV. *E*, box plot distribution of the fold current recovered after the application of diC8-PI(4,5)P_2_ with Ca^2+^ (N = 8). diC8-PI(4,5)P_2_, dioctanoyl phosphatidylinositol 4,5-bisphosphate; TMEM16A, TransMEMbrane 16A.

Endogenous TMEM16A-conducted currents were recorded from *X. laevis* oocytes in the excised inside–out patch configuration. Briefly, inside–out patch currents were recorded during 150 ms steps to −60 and +60 mV before and during application of saturating Ca^2+^. As previously reported, we observed robust Ca^2+^-activated TMEM16A-conducted currents shortly after excision (28). Figure 1*A* depicts currents recorded at −60 and +60 mV at indicated time points (10–180 s) following 2 mM Ca^2+^ addition. These currents decayed over time despite the continued Ca^2+^ application (Fig. 1, *A*–*C*). By fitting single exponential functions (Equation 1) to plots of normalized currents recorded during steps to −60 mV *versus* time, we found that the average rate of rundown was 80.6 ± 14.8 s (N = 11).

The currents of PI(4,5)P_2_-regulated channels characteristically rundown when recorded using the inside–out configuration of the patch-clamp technique. Indeed, we found that application of 100 μM of the soluble dioctanoyl phosphatidylinositol 4,5-bisphosphate (diC8-PI(4,5)P_2_) recovered TMEM16A currents (28). Figure 1*D* shows an example plot of TMEM16A-conducted currents recorded at −60 mV *versus* time, before and during application of 100 μM diC8-PI(4,5)P_2_. In nine independent trials, we observed that diC8-PI(4,5)P_2_ recovered an average of 3.5 ± 0.4-fold current (Fig. 1*E*).

**Figure 2:**
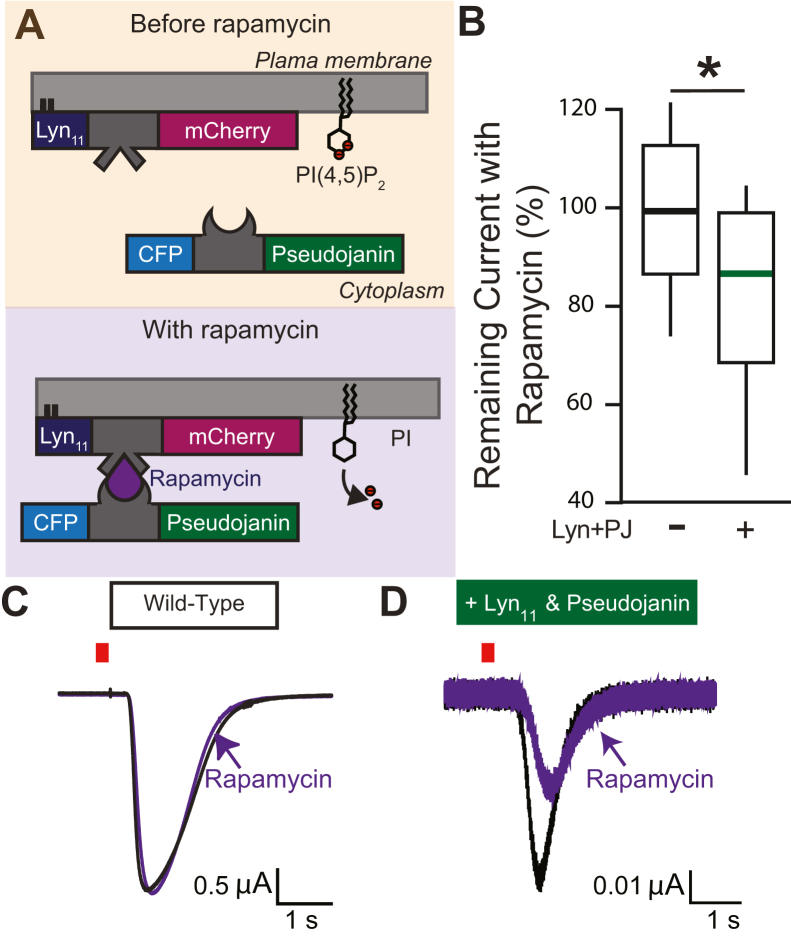
**TMEM16A Ca**^**2+**^**-evoked Cl**^−^**currents are depleted in whole *Xenopus laevis* oocytes by dephosphorylation of PI(4,5)P**_**2**_**.***A*, schematic demonstrating pseudojanin translocation to the plasma membrane. To express pseudojanin at the membrane, the membrane tether Lyn_11_-mCherry and pseudojanin-CFP RNAs were both injected into *X. laevis* oocytes. Lyn_11_-mCherry expresses at the plasma membrane, and pseudojanin expresses in the cytoplasm. Upon rapamycin application, rapamycin binds Lyn_11_-mCherry and induces the membrane translocation of pseudojanin-CFP. Once at the membrane, pseudojanin-CFP dephosphorylates PI(4,5)P_2_ at the 4′ and 5′ position. The effects of pseudojanin on whole-cell TMEM16A Ca^2+^-evoked Cl^−^ currents were measured using the two-electrode voltage-clamp technique. *B*, box plot distribution of the percentage remaining current observed in uninjected control and pseudojanin-CFP–expressing *X. laevis* oocytes after incubation in 10 μM rapamycin for 5 min. The percent of remaining currents was significantly different (*p* = 0.02) as determined by a two-tailed *t* test. ∗ denotes *p* < 0.05. *C* and *D*, example of whole-cell currents recorded at −80 mV before and after rapamycin application in oocytes expressing pseudojanin-CFP. Current was recorded in control solution (*black*) and after incubation in rapamycin for 5 min (*purple*). *Red bar* represents 250 ms duration of UV light application. CFP, cyan fluorescent protein; PI(4,5)P_2_, phosphatidylinositol 4,5-bisphosphate; TMEM16A, TransMEMbrane 16A.

To explore whether reducing membrane PI(4,5)P_2_ also depletes TMEM16A currents in whole oocytes, we employed a rapamycin-induced dimerization system to translocate a cytosolic phosphatase, pseudojanin, to a membrane-anchored domain Lyn_11_ (31) (Fig 2*A*). At the plasma membrane, the synthetic enzyme pseudojanin dephosphorylates PI(4,5)P_2_ at both the 4′ and 5′ positions (31). Using TEVC recordings, we compared how rapamycin application altered the TMEM16A-conducting currents in wildtype *X. laevis* oocytes with those expressing pseudojanin and Lyn_11_-mCherry. UV light application photoactivated caged-IP_3_ to increase intracellular Ca^2+^ in oocytes clamped at −80 mV. In wildtype oocytes, rapamycin application did not alter the TMEM16A-conducted currents; we observed an average of 101 ± 5.8% with rapamycin treatment compared with currents observed before treatment (N = 13) (Fig. 2, *B* and *C*). By contrast, we observed that in cells expressing Lyn_11_ and pseudojanin, the percent of remaining current after rapamycin-induced dimerization was an average of 80.8 ± 6.1% (N = 19, *p* = 0.02, *t* test) (Fig. 2, *B* and *D*). Together, these data demonstrate that PI(4,5)P_2_ potentiates the endogenous TMEM16A channels in *X. laevis* oocytes in both excised patches and whole cells.

### Phospholipids differentially recovered TMEM16A currents following rundown

**Figure 3:**
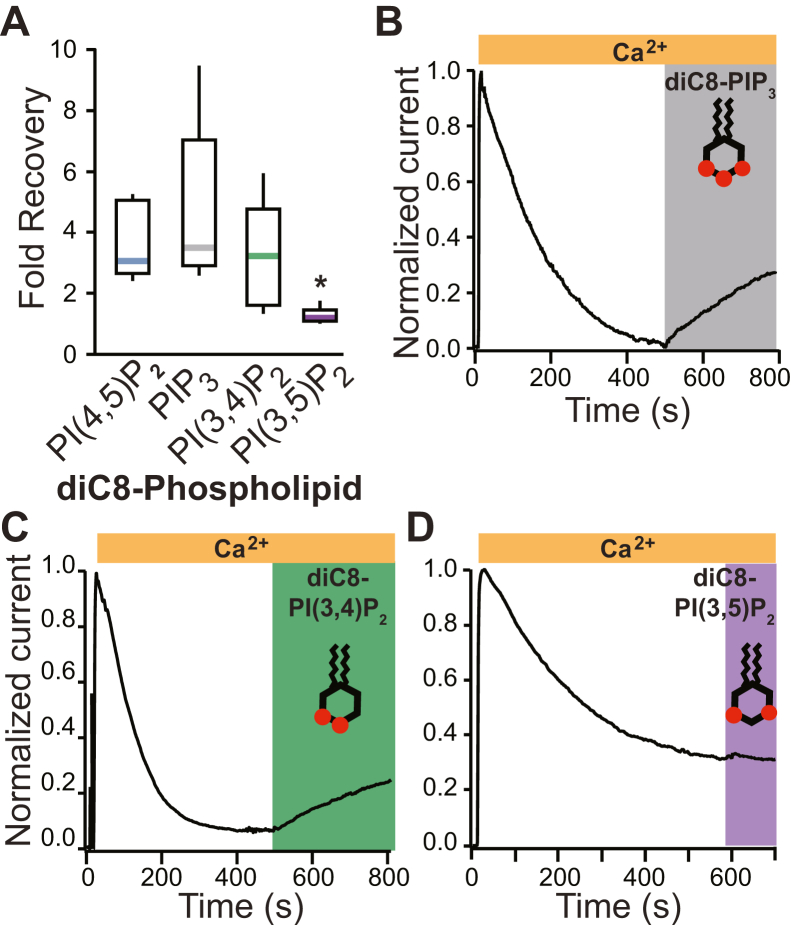
**Phospholipid analogs differentially recovered TMEM16A.** Soluble synthetic analogs of PIP_3_ (diC8-PIP_3_), PI(3,4)P_2_ (diC8-PI(3,4)P_2_), and PI(3,5)P_2_ were applied to excised inside–out patches once current had stably rundown. Currents were recorded at −60 mV. *A*, box plot distribution of the fold current recovered after the application of diC8-PI(4,5)P_2_ (N = 9), diC8-PIP_3_ (N = 7), diC8-PI(3,4)P_2_ (N = 6), or diC8-PI(3,5)P_2_ (N = 5). Representative plots of normalized currents *versus* time, before and during application of 100 μM diC8-PIP_3_ (*B*), diC8-PI(3,4)P_2_ (*C*), or diC8-PI(3,5)P_2_ (*D*). ∗ represents *p* < 0.025 as determined by ANOVA and Tukey's HSD post hoc tests. diC8-PI(3,4)P_2_, dioctanoyl phosphatidylinositol 3,4-bisphosphate; diC8-PI(3,5)P_2_, dioctanoyl phosphatidylinositol 3,5-bisphosphate; diC8-PI(4,5)P_2_, dioctanoyl phosphatidylinositol 4,5-bisphosphate; diC8-HSD, honestly significant difference; PIP_3,_ dioctanoyl phosphatidylinositol 3,4,5-trisphosphate; PI(3,4)P_2_, phosphatidylinositol 3,4-bisphosphate; PI(3,5)P_2_, dioctanoyl phosphatidylinositol 3,5-bisphosphate; PIP_3,_ phosphatidyl 3,4,5-trisphosphate; TMEM16A, TransMEMbrane 16A.

To further characterize the TMEM16A–PI(4,5)P_2_ interaction, we explored whether the number of negative charges carried by the inositol phosphate altered the ability of the lipid to regulate channel activity. We began by testing the hypothesis that applying phosphoinositol analogs with more phosphate groups, and therefore more negative charges, would be more effective at recovering TMEM16A current following rundown. We applied a water-soluble analog of dioctanoyl phosphatidylinositol 3,4,5-trisphosphate (diC8-PIP_3_) to excised inside–out patches after current had stably rundown. In seven independent trials, we observed that 100 μM diC8-PIP_3_ application recovered an average of 4.6 ± 1.0-fold current, which was not significantly different from the current recovered by diC8-PI(4,5)P_2_ (Fig. 3*A*) (*p* = 0.27, Tukey’s honestly significant difference [HSD] test). Figure 3*B* shows an example plot of TMEM16A-conducted currents *versus* time before and during diC8-PIP_3_ application. These data reveal that the phosphate groups’ negative charge is insufficient to predict how the phospholipids potentiate TMEM16A currents.

We next examined whether the relative phosphate position determined how effective a phospholipid recovered TMEM16A. We applied diC8-phospholipids that included two variably placed phosphate groups on the inositol ring. We started with another vicinal diphospholipid: dioctanoyl phosphatidylinositol 3,4-bisphosphate (diC8-PI(3,4)P_2_) (Fig. 3*C*). In six independent trials, we observed that 100 μM of diC8-PI(3,4)P_2_ recovered an average of 3.1 ± 0.7-fold current (Fig. 3, *A* and *C*), which was similar to the recovery observed with diC8-PI(4,5)P_2_ (3.5 ± 0.4-fold current, N = 8).

Not all biphosphate analogs effectively recovered TMEM16A-conducted current. In five independent trials, dioctanoyl phosphatidylinositol 3,5-bisphosphate (diC8-PI(3,5)P_2_) recovered only 1.3 ± 0.1-fold current (Fig. 3, *A* and *D*), significantly less than that recovered by diC8-PI(4,5)P_2_ (*p* < 0.01, Tukey’s HSD test). These data reveal that the number of negatively charged phosphate groups does not explain how effectively a phospholipid can recover TMEM16A-conducted current. The two phospholipids that effectively regulated TMEM16A currents shared two characteristics: they both had vicinal phosphate groups and phosphate groups at the 4′ position.

### Phosphate at position 4′ of the inositol ring is key in the interaction of PI(4,5)P_2_ with TMEM16A

**Figure 4:**
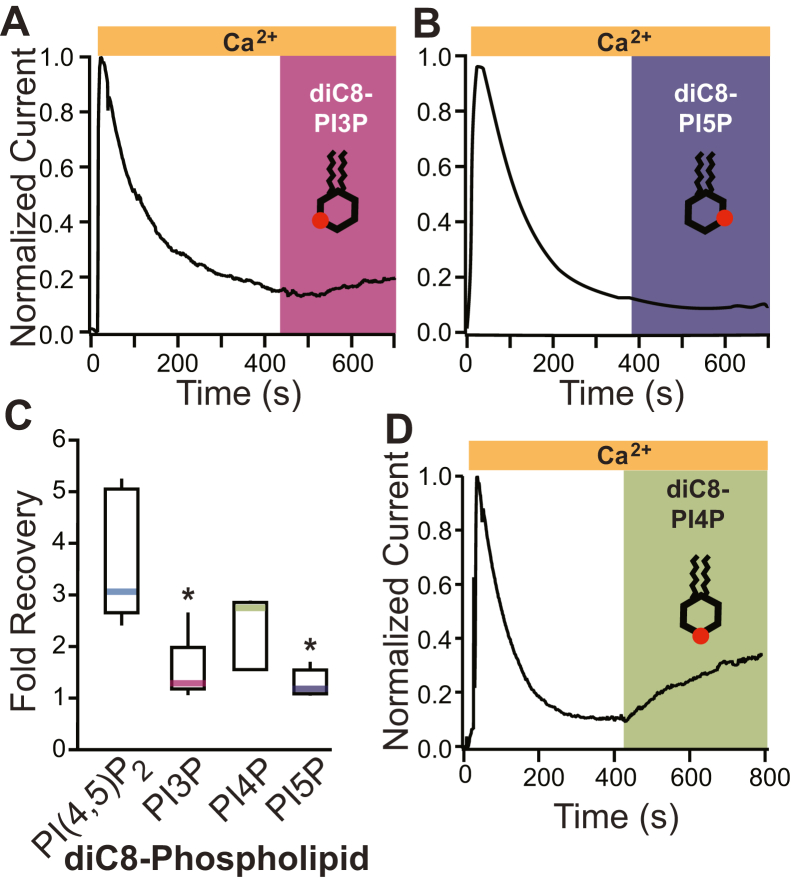
**Phospholipids with phosphates at position 4′ of the inositol ring recover current.** Soluble synthetic analogs of PI3P, PI4P, and PI5P were applied to excised inside–out patches once current had stably rundown. Currents were recorded at −60 mV. Representative plots of normalized currents *versus* time, before and during application of 100 μM diC8-PI3P (*A*), 100 μM diC8-PI5P (*B*), or 100 μM diC8-PI4P (*D*). *C*, box plot distribution of the fold current recovered after the application of diC8-PI(4,5)P_2_ (N = 9), diC8-PI3P (N = 7), diC8-PI4P (N = 7), or diC8-PI5P (N = 5). ∗ denotes *p* < 0.05 between indicated treatment and diC8-PI(4,5)P_2_, as determined by ANOVA and Tukey’s HSD post hoc tests. diC8-PI3P, dioctanoyl 3-monophosphate; diC8-PI4P, dioctanoyl 4-monophosphate; diC8-PI5P, dioctanoyl 5-monophosphate; HSD, honestly significant difference.

We predicted that if vicinal phosphates were required to potentiate TMEM16A, then diC8 analogs with a single phospholipid should not recover TMEM16A currents following rundown. By contrast, if a phosphate at the 4′ position is required to potentiate TMEM16A-conducted current, only dioctanoyl 4-monophosphate (diC8-PI4P), but not dioctanoyl 3-monophosphate (diC8-PI3P) or dioctanoyl 5-monophosphate (diC8-PI5P), should recover TMEM16A currents. To discriminate between these possibilities, we applied phosphoinositol monophosphates to excised patches following rundown. We first assessed if phosphates at positions 3′ and 5′ of the inositol head would also recover TMEM16A following rundown. Figure 4, *A* and *B* shows example plots of TMEM16A-conducted currents recorded at −60 mV *versus* time, before and during diC8-PI3P or diC8-PI5P application. We observed that the application of 100 μM diC8-PI3P recovered 1.5 ± 0.3-fold current (N = 5) and that diC8-PI5P only recovered 1.3 ± 0.1-fold current (Fig. 4, *A*–*C*).

Next, we assessed if the phosphate at position 4′ was indeed important for phosphoinositol potentiation of TMEM16A-conducted Cl^−^ currents. We applied diC8-PI4P to excised inside–out patches after the current had stably rundown. We recorded TMEM16A-conducted currents at −60 mV before and during the application of 100 μM diC8-PI4P. In seven separate trials, we observed that 100 μM diC8-PI4P application recovered 2.4 ± 0.9-fold current (Fig. 4, *C* and *D*). The currents recovered by diC8-PI4P were similar albeit smaller than the 3.5 ± 0.4-fold recovery observed with diC8-PI(4,5)P_2_ (*p* > 0.1, Tukey’s HSD test) (Fig. 4*C*). Together, these data were consistent with the hypothesis that a phosphate group at the 4′ position was critical for the phospholipid to recover TMEM16A-conducted currents.

**Figure 5:**
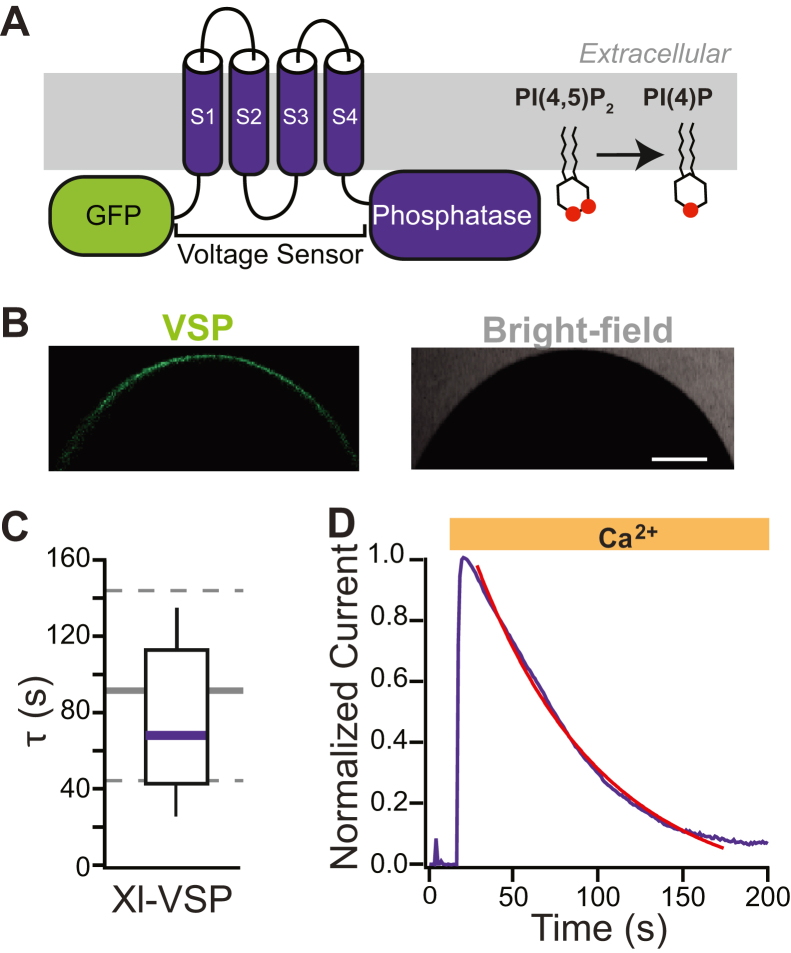
**Xl-VSP does not significantly change TMEM16A current rundown.** Inside–out patch-clamp recordings were conducted on macropatches excised from *Xenopus laevis* oocytes expressing Xl-VSP. *A*, schematic depicting a VSP tagged with GFP. *B*, confocal and bright-field images of a representative *X. laevis* oocyte expressing GFP-tagged Xl-VSP at the plasma membrane. Bar denotes 200 μm. *C*, box plot distribution of the rate of current decay (τ), measured by fitting plots of relative current *versus* time with single exponentials for the Xl-VSP expressing (*purple*) (N = 6). Background *gray dashed lines* denote 25 to 75% of the data spread, and the *solid line* represents the median rate of rundown measured from patches recorded under the control conditions (plotted in Fig. 1*C*). *D*, representative plot of normalized currents *versus* time following VSP activation. TMEM16A, TransMEMbrane 16A; Xl-VSP, *Xenopus laevis* voltage-sensing phosphatase.

As another test of the importance of a 4′ phosphate, we exogenously expressed a VSP in *X. laevis* oocytes and used membrane depolarization to activate dephosphorylation of PI(4,5)P_2_ at the 5′ position in excised patches (Fig. 5*A*). If the 4′ phosphate is required for the phospholipid to potentiate TMEM16A channels, then activation of VSP should not speed rundown. By contrast, if vicinal phosphates are required for the lipid to regulate these currents, then VSP activation should speed rundown. We used the VSP endogenously expressed in *X. laevis* sperm (Xl-VSP) with a GFP on the N terminus. GFP fluorescence confirmed VSP expression (Fig. 5*B*) prior to patch clamp recordings. Notably, the Xl-VSP enzyme is turned on by voltages more positive than −20 mV, with maximal activation observed at +40 mV (32). We focused on oocytes with GFP fluorescence and recorded TMEM16A Ca^2+^-evoked Cl^−^ currents. In five independent trials, we recorded TMEM16A-conducted currents at −60 mV before and during the application of Ca^2+^ in patches while activating Xl-VSP at depolarizing potentials (+60 mV). We observed that the rate of current rundown in patches expressing Xl-VSP at the membrane was 76.1 ± 18.1 s and was not significantly different compared with the control-current rundown (80.5 ± 14.8 s; N = 11; Fig. 5*C*, *t* test, *p* = 0.63). Figure 5*D* shows an example plot of TMEM16A-conducted currents recorded at −60 mV *versus* time with Xl-VSP activation. The rate of TMEM16A-current rundown is similar in the presence and absence of VSP, consistent with the hypothesis that the 4′ phosphate is critical for phospholipid regulation of TMEM16A-channel activity.

## Discussion

This study sought to uncover how PI(4,5)P_2_ interacts with TMEM16A channels. As we have reported previously (28), we again found that Ca^2+^-activated TMEM16A-conducted currents rundown over time when recorded in the inside–out configuration of the patch-clamp technique, despite the continued application of Ca^2+^. We previously reported that TMEM16A-conducted currents rundown in excised patches because of continued activity of membrane-associated phosphatases that dephosphorylate the inositol head group of PI(4,5)P_2_, without the activity of counteracting kinases (28). We report that application of the water-soluble phospholipid diC8-PI(4,5)P_2_ recovered these currents. Here, we in addition report that degradation of PI(4,5)P_2_ in whole *X. laevis* oocytes also reduced TMEM16A-conducted currents. By exogenously expressing the soluble phosphatase pseudojanin and the membrane-tethered N terminus of Lyn kinase (Lyn_11_), we induced dephosphorylation of PI(4,5)P_2_ by applying rapamycin. Because the unphosphorylated PI does not potentiate TMEM16A-conducted currents (28), we predicted that rapamycin application should decrease TMEM16A-conducted current in oocytes expressing both pseudojanin and Lyn_11_. Indeed, we observed a rapamycin-evoked reduction in the averaged current only in oocytes with pseudojanin and Lyn_11_. There were, however, differences observed amongst oocytes injected with the pseudojanin and Lyn_11_ circular RNA (cRNA); rapamycin exerted no measurable change in some trials but reduced the current by 95% in others (Fig. 2*B*). This variability could result from differences in expression of both membrane-anchored Lyn_11_ and soluble pseudojanin.

Having validated the importance of PI phosphorylation in PI(4,5)P_2_–TMEM16A interactions (28), we sought to identify the determinants of this interaction. We found that various phosphoinositides applied to inside–out patches differentially recovered TMEM16A-conducted currents following rundown. Comparing only bisphosphates and trisphosphates, we found that diC8-PI(4,5)P_2_, diC8-PI(3,4)P_2_, and diC8-PI(3,4,5)P_3_ each effectively recovered TMEM16A-conducted currents following rundown; by contrast, diC8-PI(3,5)P_2_ did not (Fig. 3). The inability of this phospholipid to recover current is consistent with two hypotheses: that the PI(4,5)P_2_–TMEM16A interaction involves TMEM16A binding to vicinal phosphate groups or that binding requires a phosphate group at the 4′ position of the PI inositol ring.

To discriminate between these possibilities, we probed for current recovery by lipids with single phosphate groups. We found that only diC8-PI4P significantly recovered TMEM16A-conducted current following rundown (Fig. 4). The inability of the 3′ and 5′ monophospholipids to potentiate TMEM16A was strikingly similar to the inability of diC8-PI to recover current, as we have previously reported (28). Together, these data reveal that the 4′ phosphate is a key determinant of TMEM16A regulation by PIPs. The individual contributions of the monophosphates are not additive for currents recovered by PI(4,5)P_2,_ and the extent of current recovery depends on whether a phosphate is present at position 4′ of the inositol head (Figs. 3 and 4).

We observed that the rate of current recovery with diC8-lipid application varied between experiment trials. These differences may reflect variation in the speed of lipid application or the shape of the patch between experimental trials.

As an alternate approach to test whether a 4′ phosphate group is sufficient to mediate TMEM16A gating, we probed whether removing the 5′ phosphate altered the rundown of TMEM16A-conducted currents by expressing Xl-VSP, the VSP originally cloned from *X. laevis* sperm. We chose this phosphatase because it has been successfully expressed in *X. laevis* oocytes (33), and it is activated at less depolarizing potentials (−20 mV) compared with other commonly used VSPs from the tunicate *Ciona intestinalis* (0 mV) or zebrafish (+50 mV) (33, 34). To remove the 5′ phosphate, we held excised patches at 0 mV and stepped to +60 mV to turn on the VSP. We predicted that if the 4′ position was key, activating exogenously expressed Xl-VSP should not significantly alter rundown. We did not observe a significant difference between the rate of rundown in wildtype and Xl-VSP–expressing patches (Fig. 5), thereby supporting our hypothesis that the 4′ phosphate is critical for PI(4,5)P_2_ regulation of TMEM16A-conducted currents.

Our finding that PI(4)P is sufficient to potentiate TMEM16A is surprising given that previous studies in mouse TMEM16A have demonstrated that the 4′ phosphate is dispensable (26). Still others have found that VSP activation does successfully diminish TMEM16A-conducted currents (35).

**Figure 6:**
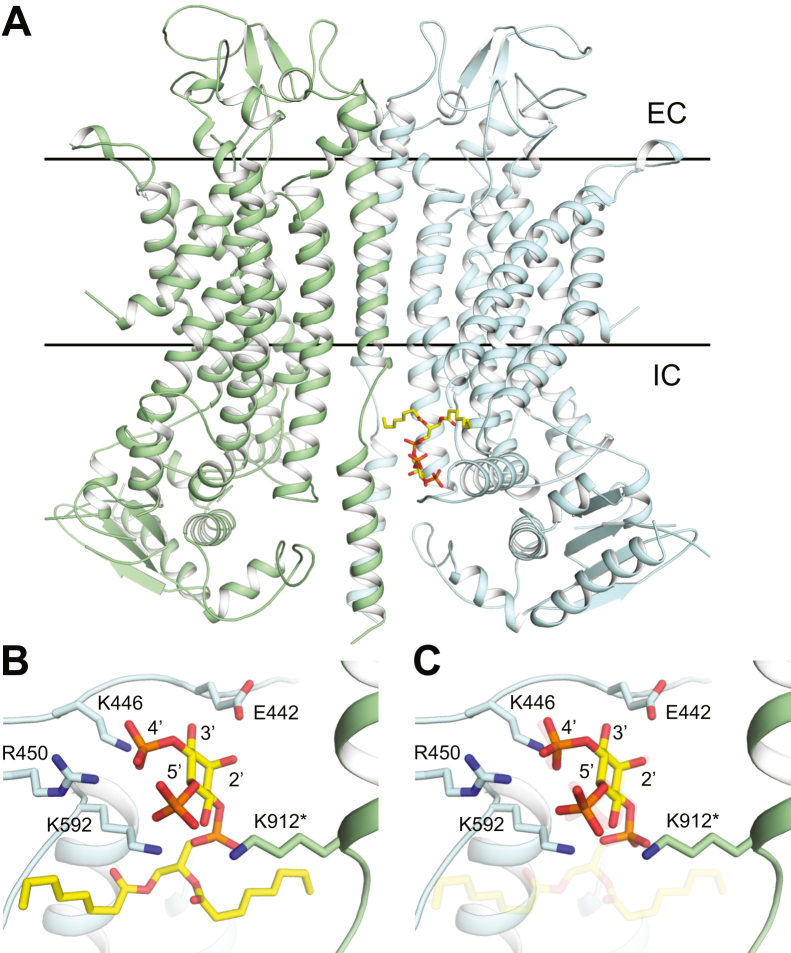
**Docking suggests key PI(4,5)P**_**2**_**phosphate interactions with TMEM16A.** Docking was performed with either diC8-PI(4,5)P_2_ or IP_3_ into a homology model of *Xenopus laevis* TMEM16A (xTMEM16A). *A*, position of diC8-PI(4,5)P_2_ shown against the homology model of xTMEM16A. *Lines* indicating the position of the intracellular and extracellular boundaries of the plasma membrane were created using the OPM entry for mouse TMEM16A (PDB: 5OYB). *B*, detailed view of the hypothesized PI(4,5)P_2_–xTMEM16A interaction. Interacting residues (E442, K446, R450, K592, and K912 from the other chain) and phosphates (positions 2′–5′) are highlighted. *C*, superposition of docked IP_3_ (foreground) on the PI(4,5)P_2_–xTMEM16A (transparency) interaction. diC8-PI(4,5)P_2_, dioctanoyl phosphatidylinositol 4,5-bisphosphate; PDB, Protein Data Bank; PI(4,5)P_2,_ dioctanoyl phosphatidylinositol 4,5-bisphosphate; TMEM16A, TransMEMbrane 16A; xTMEM16A, *Xenopus laevis* TransMEMbrane 16A.

We attempted to resolve these conflicting findings by performing computer docking of PI(4,5)P_2_ into a homology model of *X. laevis* TMEM16A (Fig. 6*A*). The modeled region (residues 113–931) of *X. laevis* TMEM16A is 82.2% identical to the corresponding region in the mouse channel and aligns to mouse TMEM16A with an RMSD of 1.1 Å (Fig. S1*A*). Notably, our model preserves putative PI(4,5)P_2_-binding sites identified in mouse TMEM16A (26, 29) including a strongly basic cluster formed between TM5 and the TM2–3 linker. Docking diC8-PI(4,5)P_2_ into the *X. laevis* TMEM16A homology model suggests that the 4′ phosphate forms a salt bridge with the conserved residue K446 (K451 in the mouse TMEM16A) (Fig. 6*B*). Notably, K446 is part of the EAVK motif also known as the c-segment (exon 13) of mouse TMEM16A. This motif is removed in a splice variant (EAVK, Δ448–451, in the linker connecting transmembrane domains 2 and 3) of the mouse channel (36). The ΔEAVK splice variant was used in a previous study reporting that PI4P did not potentiate TMEM16A-conducted currents (26). This difference is notable because the hypothesized PI(4,5)P_2_-binding sites are otherwise nearly identical (Fig. S1*B*). Other studies have shown that mutating K451 of the EAVK motif to alanine abrogates PI(4,5)P_2_ dependence in mouse TMEM16A, suggesting that PI(4,5)P_2_ interactions with TMEM16A are modulated by alternative splicing (35). Although ΔEAVK has been identified in other organisms, including humans, where it constitutes a minority of TMEM16A transcripts in the brain and skeletal muscle (36, 37), no evidence exists for alternative TMEM16A splice variants in *X. laevis*. We also performed docking with IP_3_, which yielded a nearly superimposable binding pose as found for diC8-PI(4,5)P_2_ (Fig. 6*C*) and substantiated the head group contacts identified for PI(4,5)P_2_.

The *X. laevis* TMEM16A model includes gaps, notably one (461–507), near the docked diC8-PI(4,5)P_2_ moiety. We assessed the possibility that this missing region might interfere with diC8-PI(4,5)P_2_ docking by superimposing the AlphaFold-generated model of mouse TMEM16A (38), which includes these regions, onto our homology model (Fig. S1*C*). AlphaFold predicts that the missing gap is a disordered loop. The predicted loop conformation is not grossly incompatible with our predicted diC8-PI(4,5)P_2_–binding pose, although some minor side-chain adjustments would be necessary to accommodate the ligand.

The required 4′ phosphate may support a previously proposed networked PI(4,5)P_2_-binding model for mouse TMEM16A (29). In this model, TMEM16A contains three PI(4,5)P_2_-binding sites, formed by basic residues that, when neutralized, reduce PI(4,5)P_2_ sensitivity (29). These sites are conserved in *X. laevis*, and the 4′ phosphate may be required for binding one or more of these sites. However, this model does not account for the apparent contribution of mouse TMEM16A residue K451 in binding PI(4,5)P_2_, as the proposed PI(4,5)P_2_ sites are located elsewhere (29, 35, 39). One possibility is that our proposed binding site (Fig. 6*B*) works to allosterically regulate the activation of TMEM16A. Further study is needed to resolve this intriguing hypothesis.

## Experimental procedures

### Reagents

The following dioctanoyl phospholipids were obtained from Echelon Biosciences: diC8-PI3P, diC8-PI4P, diC8-PI5P, diC8-PI(3,4)P_2_, diC8-PI(4,5)P_2_, diC8-PI(3,5)P_2_, and diC8-PIP_3_. Plasmids encoding Lyn_11_-targeted FRB and pseudojanin were purchased from Addgene (40). Unless otherwise noted, all other reagents were purchased from Thermo Fisher Scientific.

### Animals

Animal procedures used were approved by the Animal Care and Use Committee at the University of Pittsburgh and were consistent with the accepted standards of humane animal care. *X. laevis* adult oocyte-positive females were obtained commercially (NASCO) and housed at 18 °C with a 12/12-h light/dark cycle.

### Oocyte collection

*X. laevis* females were anesthetized by immersion in 1.0 g/l tricaine (pH 7.4) for 30 min before oocytes were collected. The ovarian sacs were surgically obtained and manually pulled apart using blunt forceps. Oocytes were treated with a 90-min incubation in 1 mg/ml collagenase in the ND96 solution and then repeatedly rinsed in oocyte Ringer's solution 2 (OR2) to remove collagenase. Healthy oocytes were stored at 14 °C in pyruvate- and gentamycin-supplemented ND96 solution.

### Solutions

Inside–out patch-clamp recordings were conducted in a Hepes-buffered saline solution made as follows (in millimolar): 130 NaCl and 3 Hepes, pH 7.2, and filtered using a sterile 0.2 μm polystyrene filter (28). The Hepes-buffered saline solution was supplemented with 0.2 μM EGTA for Ca^2+^-free recordings. For recordings made with intracellular Ca^2+^, the Hepes-buffered saline solution was supplemented with 2 mM CaCl_2_ with indicated reagents. For current recovery experiments, the diC8 analogs were added to the Ca^2+^-containing Hepes-buffered saline solution and applied as indicated. TEVC recordings were conducted in solution ND96 made as follows (in millimolar): 96 NaCl, 2 KCl, 1 MgCl_2_, 10 Hepes, pH 7.6. The solution was filtered with a sterile 0.2-μm polystyrene filter (16).

The oocyte wash solution, called OR2, and storage solution, ND96, were made as follows. OR2 (in millimolar): 82.5 NaCl, 2.5 KCl, 1 MgCl_2_, and 5 mM Hepes, pH 7.2. ND96 (in millimolar): OR2 supplemented with 5 mM sodium pyruvate, 100 mg/l gentamycin, pH 7.6, and 0.2 μm polystyrene filtered.

### Patch-clamp recordings

Patch-clamp recordings were made on *X. laevis* oocytes after manually removing the vitelline membrane. TMEM16A current recordings made in the inside–out configuration of the patch-clamp technique (41) used an EPC-10 USB patch-clamp amplifier (HEKA Elektronik). Data were acquired with Patchmaster software (HEKA Elektronik). Briefly, upon formation of a gigaseal (greater than 1 GΩ), inside–out patches were excised in Hepes-buffered saline solution lacking both EGTA and added CaCl_2_. In this EGTA-free Hepes-buffered saline, the patch resistances often decreased to 20 to 200 MΩ following excision but returned to greater than 1 GΩ with EGTA application. Data were collected at a rate of 10 kHz. Glass pipettes were pulled from borosilicate glass (outer diameter = 1.5 mm; inner diameter = 0.86 mm; Warner Instruments) and fire polished (Narishige Microforge). Each pipette had a resistance of 0.4 to 1.5 MΩ. All data collection for inside–out patch experiments was initiated within 10 s of patch excision. All diC8 lipid analogs were applied to excised inside–out patches in an RC-28 chamber (Warner Instruments). Other solutions were applied to excised patches using a VC-8 fast perfusion system (Warner Instruments).

Patch-clamp data were analyzed with Excel (Microsoft) and IGOR Pro (Wavemetrics) with Patchers Power Tools. Currents were processed such that peak currents obtained with 2 mM intracellular Ca^2+^ application were normalized to 1. The basal currents recorded once the patch reached a steady state following rundown.

To calculate the rate of rundown, plots of normalized current at −60 mV *versus* time are fit with the single exponential equation:

(1) $$Y\left(x\right)=\;Y_{0}{\text{e}}^{\frac{-x}{t}}$$

where *Y*_0_, *x*, *τ*, and *Y*(*x*) represent the initial current, time, rate of current rundown, and current at time *x* (28).

To compare the current recovered following application of different synthetic lipid analogs, the fold change in current recovered was calculated by dividing the peak current after diC8-analog addition by the baseline current. The peak current was defined as the highest current obtained after diC8-analog addition. The baseline current was defined as the current observed at the point of diC8-analog addition. The equation used was:

(2) $$\text{Fold recovery}=\frac{\text{Maximum current after analog addition}}{\text{Baseline current at analog addition}}$$

A fold recovery of one relates to unchanged current, and a recovery >1 indicates that diC8-analog application increased the current.

Each experimental condition includes trials conducted on multiple days with oocytes collected from different females.

### Exogenous protein expression in *X. laevis* oocytes

The complementary DNAs encoding the Lyn_11_ and pseudojanin constructs were engineered into the GEMHE vector using overlapping extension PCR. The sequences for all constructs were verified by automated Sanger sequencing (Genewiz). The Xl-VSP construct was provided by L. Jaffe (University of Connecticut (32)). The cRNAs for these constructs were transcribed using the T7 mMessage mMachine Ultra kit (Ambion). Defolliculated oocytes were injected with 5 ng of cRNA for both Lyn_11_ and pseudojanin or Xl-VSP. Injected oocytes were screened for Lyn_11_-mCherry or Xl-VSP-GFP expression at the membrane using confocal imaging 48 h after cRNA injection.

### TEVC recordings

TEVC recordings were made using TEV-200A amplifiers (Dagan Co) and digitized by Axon Digidata 1550A (Molecular Devices). Data were acquired with pClamp Software (Molecular Devices) at a rate of 5 kHz. Recordings were made on *X. laevis* oocytes 48 h after cRNA injection and 1 to 4 h after injection of photolabile IP_3_ analog myoinositol 1,4,5-trisphosphate, P4(5)-1-(2-nitrophenyl) ethyl ester (caged IP_3_).

IP_3_-evoked currents were recorded in the TEVC configuration at −80 mV from *X. laevis* oocytes. Oocytes were injected with a 200 μM caged IP_3_ stock made in double-distilled water to reach a final concentration of 5 μM within the oocyte and incubated in the dark at 18 °C before recording (10). Pipettes of 1 to 8 MΩ resistance were pulled from borosilicate glass and filled with 1 M KCl. A 250-ms exposure to UV light (Ultra High Power White LED Illuminator, 380-603; Prizmatix) was used to release the nitrophenyl cage on IP_3_. A liquid light source guided the light to oocytes in the recording chambers (RC-26G; Warner Instruments). The solution bathing the oocytes was changed with the gravity-fed pinch valve VC-8 solution changer (Warner Instruments). It is impossible to directly compare current amplitudes generated in different oocytes because of the innate variability of the experimental setup (*e.g.*, positioning of the UV light or the exact amount of caged IP_3_ in each oocyte). Changes in the background-subtracted peak currents were quantified from two consecutive recordings. The difference between the peaks of these consecutive recordings was used to calculate the remaining current (%).

To compare the effects of rapamycin on TMEM16A currents in wildtype oocytes, or oocytes expressing pseudojanin and Lyn_11_, we first normalized the currents observed by subtracting baseline currents from peak currents for both currents measured. Then, we calculated the percent change in current after rapamycin treatment relative to before treatment. The percent change was plotted as the proportion of remaining current observed with rapamycin.

We sought to express a channel whose currents would diminish with VSP as a positive control for the Xl-VSP experiments. VSP activation reportedly diminished currents from Kir2.1 channel (42). Here, we expressed Kir2.1 in *X. laevis* oocytes (Addgene plasmids 32669 and 32641, (43, 44)) and used TEVC to record whole-cell currents while ramping the voltage from −150 to +50 mV, over 2 s. Recording in a potassium-based solution comprised of (in millimolar): 90 KCl, 5 Hepes, 0.1 CaCl_2_, 1 MgCl_2_, pH 7.6 (45). We did not observe rectification from either channel.

### Quantification and statistical analyses

Data for each experimental condition are reported as mean ± SEM values and displayed in Tukey's box plot distributions. Tukey's box plot displays the data between 25 and 75 percentile, and the whiskers span 10 to 90. Phosphoinositide current recovery was analyzed using ANOVAs followed by post hoc Tukey's HSD tests to compare each phosphoinositide to diC8-PI(4,5)P_2_. Two-tailed *t* tests were used to determine differences between Xl-VSP and control patch-clamp recordings and pseudojanin currents in rapamycin *versus* wildtype oocytes treated with rapamycin for TEVC recordings.

### Imaging

*X. laevis* oocytes were imaged using a TCS SP5 confocal microscope (Leica Microsystems) equipped with a Leica 506224 5× objective. The mCherry fluorophore was excited with a 561-nm laser, and GFP was excited with a 488-nm visible laser. Using a galvo-scanner with a unidirectional (600 Hz) scanning, sequential frames were captured with 2× line averaging. Images were analyzed using LAS AF (version 2.7.3; Leica) software.

### Homology modeling

To identify a suitable template for homology modeling, we performed a BLAST search of the Protein Data Bank (46) using the UniProt B5SVV6 sequence (*X. laevis* TMEM16A) (47). The search identified a dimeric structure of *Mus musculus* TMEM16A (Protein Data Bank ID: 7B5D (48); 76.9% identity with *X. laevis* TMEM16A), resolved using cryo–electron microscopy at 3.30 Å. We ran the 7B5D structure through Schrödinger Maestro’s Protein Preparation Wizard using the default parameters, except we changed all selenomethionines to methionines, filled in any missing side chains, deleted all water molecules, and did not perform a final restrained minimization.

We then used the Maestro Homology Modeling module to generate a homology model of *X. laevis* TMEM16A based on the prepared 7B5D template. We performed knowledge-based modeling using the multiple templates (homomultimer) option to build a dimeric (rather than monomeric) model. We ran the resulting homology model through the Protein Preparation Wizard. We again used the default parameters, except we capped the terminal ends, changed all selenomethionines to methionines, filled in any missing side chains, and deleted all water molecules. We also performed a final restrained minimization.

### Docking

We used Schrödinger Maestro to prepare receptor grids for docking (default parameters). Each docking grid was centered on residues K446, K592, and R450. Given that TMEM16A is a homodimer, there are two such sites. We prepared separate receptor grids for each site.

To prepare small-molecule models of diC8-PI(4,5)P_2_ and IP_3_ for docking, we first obtained the SMILES strings of these two compounds from PubChem (49, 50). We then used Maestro’s LigPrep module (default parameters) to generate multiple compound models, as required to account for alternative protonation states. LigPrep produced 11 distinct ligand models: four models of PI(4,5)P_2_ and seven models of IP_3_.

Finally, we used Maestro’s Glide XP docking program (default parameters) (51, 52, 53, 54) to position each of the 11 small-molecule models within the candidate binding site associated with each of the two monomers (22 docking runs total).

### Docked-pose filtering

Computer docking suggested several candidate PI(4,5)P_2_- and IP_3_-binding poses. We used the following criteria to identify the most plausible: discard poses that do not predict reasonable interactions with K446, K592, and R450 (26, 35, 39); discard poses that position any ligand phosphate near the E442 carboxylate; discard poses that are not common to both PI(4,5)P_2_ and IP_3_, based on the assumption that the binding mechanisms of both ligands are similar; discard poses that orient the PI(4,5)P_2_ tail away from the bilayer, based on the assumption that the tail is embedded in the lipid bilayer (55, 56).

### Docking visualization

We used PyMOL (Schrödinger, Inc) to visualize all structural models and generate figures. The position of the plasma membrane was retrieved from the OPM Database (57) using 5OYB (23) as a template.

## Data availability

The xTMEM16A (*X. laevis* TransMEMbrane 16A) homology model will be shared upon request (please contact Joel Rosenbaum: jcr80@pitt.edu). All other data are included within the article.

## Supporting information

This article contains supporting information.

## Conflict of interest

The authors declare that they have no conflicts of interest with the contents of this article.
