# Supplementary material for: Phosphate position is key in mediating transmembrane ion channel TMEM16A–phosphatidylinositol 4,5-bisphosphate interaction
Source: J Biol Chem. 2022 Jul 14;298(8):102264. doi: 10.1016/j.jbc.2022.102264 (PMC9396059; doi:10.1016/j.jbc.2022.102264)
Supplement: Supplemental Figure 1 [file mmc1.pdf]

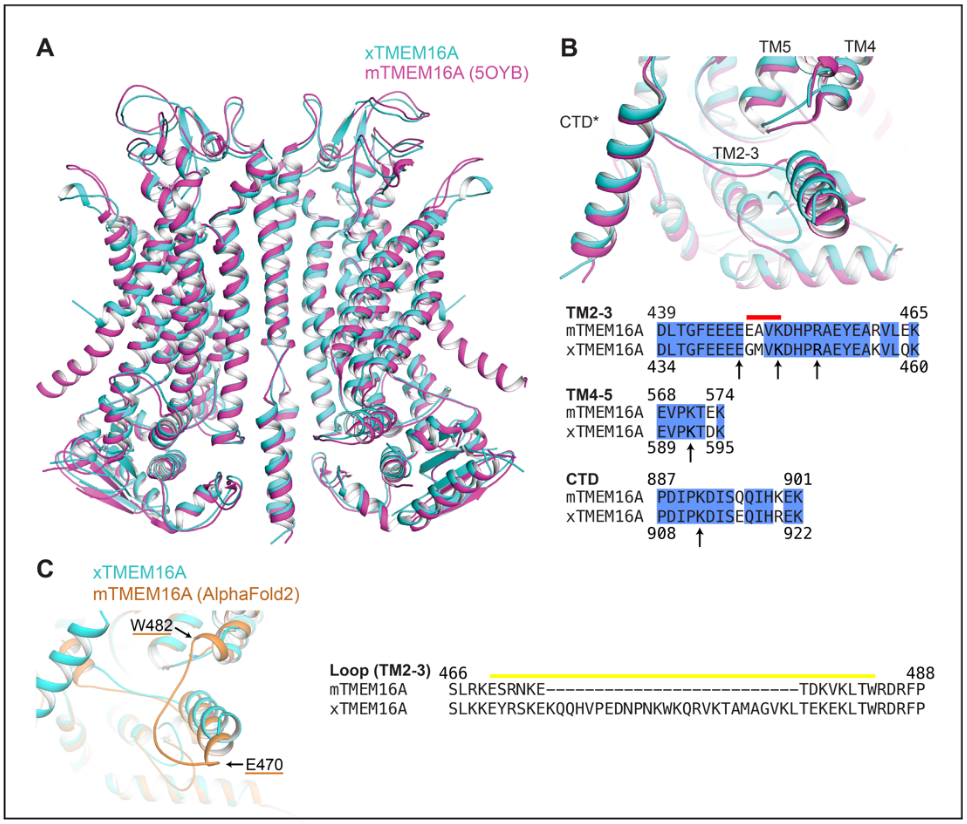

Supplemental Figure 1. **Homology model of xTMEM16A**.

A homology model of *X. laevis* TMEM16A (xTMEM16A) is highly similar to experimental structures of mouse TMEM16A (mTMEM16A, PDB 5OYB). (A) Structural alignment of xTMEM16A (cyan) and mTMEM16A (magenta). (B) Structural and sequence alignment of the hypothesized PI(4,5)P_2 binding site in xTMEM16A and mTMEM16A. Arrows highlight proposed interacting residues. The mTMEM16A (ΔEAVK) splice site is indicated by a red bar. (C) Modeling a missing loop region in the TM2-3 linker (mTMEM16A, orange). Alignment of this missing region with the xTMEM16A sequence reveals an insertion in xTMEM16A. Loop residues are indicated by a yellow bar.
